# Correction: Spatially Extensive Standardized Surveys Reveal Widespread, Multi-Decadal Increase in East Antarctic Adélie Penguin Populations

**DOI:** 10.1371/journal.pone.0165989

**Published:** 2016-10-28

**Authors:** The PLOS ONE Staff

## Notice of Republication

This article was republished on December 17, 2015, to correct errors in Fig 2, 3, and 4 that were introduced during the typesetting process. The publisher apologizes for the errors. Please download this article again to view the correct version. The originally published, uncorrected article and the republished, corrected article are provided here for reference.

## Supporting Information

S1 File Originally published, uncorrected article. (PDF)

S2 File Republished, corrected article. (PDF)
