# Supplementary material for: Spatially Extensive Standardized Surveys Reveal Widespread, Multi-Decadal Increase in East Antarctic Adélie Penguin Populations
Source: PLoS One. 2015 Oct 21;10(10):e0139877. doi: 10.1371/journal.pone.0139877 (PMC4619065; doi:10.1371/journal.pone.0139877)
Supplement: S1 File — (DOC) [file pone.0139877.s001.doc]

**Table 1. Latitude and longitude of 99 breeding sites used to estimate Adélie penguin population change in East Antarctica over the past 30 years. The 72 sites used to estimate decadal-scale change in five regional populations (S: Syowa; M: Mawson; D: Davis; C: Casey and DD: Dumont d’Urville) are marked with an a**sterisk.

| S, -69.143, 39.402* | M, -67.549, 62.979* | D, -68.829, 77.696 | D, -68.538, 77.957* | DD, -66.625, 139.725* |
| --- | --- | --- | --- | --- |
| S, -69.218, 39.420* | M, -67.790, 66.721 | D, -68.859, 77.703 | D, -68.556, 78.283* | DD, -66.631, 139.732* |
| S, -69.018, 39.442* | D, -69.007, 76.976 | D, -68.902, 77.715 | D, -68.556, 78.283* | DD, -66.638, 139.824* |
| S, -69.381, 39.473* | D, -69.026, 76.873 | D, -68.771, 77.744 | D, -68.419, 78.333* | DD, -66.679, 139.834* |
| S, -69.023, 39.490* | D, -69.033, 76.856 | D, -68.800, 77.746 | D, -68.397, 78.401* | DD, -66.684, 139.906* |
| S, -69.461, 39.683* | D, -69.036, 76.823 | D, -68.766, 77.754 | D, -68.393, 78.406* | DD, -66.691, 139.907* |
| S, -69.195, 39.687* | D, -69.041, 76.816 | D, -68.808, 77.842 | D, -68.374, 78.416* | DD, -66.691, 139.923* |
| S, -69.195, 39.687* | D, -69.046, 76.847 | D, -68.765, 77.898 | D, -68.357, 78.536* | DD, -66.673, 139.945* |
| M, -67.623, 62.472* | D, -69.135, 76.747 | D, -68.649, 77.821* | D, -68.358, 78.542* | DD, -66.672, 139.963* |
| M, -67.604, 62.479* | D, -69.136, 76.738 | D, -68.635, 77.823* | C, -66.230, 110.184* | DD, -66.659, 139.974* |
| M, -67.601, 62.481* | D, -69.147, 77.270 | D, -68.646, 77.828* | C, -66.321, 110.402* | DD, -66.671, 139.997* |
| M, -67.574, 62.491* | D, -69.148, 77.267 | D, -68.664, 77.837* | C, -66.329, 110.411* | DD, -66.670, 140.000* |
| M, -67.611, 62.501* | D, -69.149, 77.267 | D, -68.636, 77.858* | C, -66.413, 110.463* | DD, -66.665, 140.002* |
| M, -67.632, 62.513* | D, -69.334, 75.574 | D, -68.659, 77.870* | C, -66.303, 110.483* | DD, -66.671, 140.004* |
| M, -67.605, 62.539* | D, -69.337, 76.575 | D, -68.616, 77.870* | C, -66.282, 110.490* | DD, -66.670, 140.011* |
| M, -67.591, 62.817* | D, -69.338, 76.566 | D, -68.578, 77.871* | C, -66.373, 110.542* | DD, -66.674, 140.011* |
| M, -67.577, 62.884* | D, -69.343, 76.575 | D, -68.548, 77.892* | C, -66.250, 110.566* | DD, -66.667, 140.020* |
| M, -67.578, 62.894* | D, -68.866, 77.566 | D, -68.543, 77.908* | C, -66.250, 110.566* | DD, -66.663, 140.023* |
| M, -67.561, 62.929* | D, -68.879, 77.597 | D, -68.607, 77.914* | C, -66.217, 110.606* | DD, -66.645, 140.043* |
| M, -67.553, 62.965* | D, -68.840, 77.638 | D, -68.504, 77.956* | C, -66.217, 110.643* |  |

**Table 2. Sources of historical population count data**

| **Regional**  **population** | **Reference** |
| --- | --- |
| Syowa | Kato, A. and Y. Ropert-Coudert. 2006. Rapid increase in Adélie penguin populations in the Lutzow-Holm Bay area since the mid 1990s. Polar Bioscience **20**:55-62. |
| Mawson | Horne, R. S. C. 1983. The distribution of penguin breeding colonies on the Australian Antarctic Territory, Heard Island, the MacDonald Islands, and Macquarie Island. Australian Antarctic Division, Hobart. |
|  | Alonso, J. C., G. W. Johnstone, M. Hindell, P. Osborne, and R. Guard. 1987. Las aves del Monolito Scullin, Antartida Oriental (67o47'S, 66o42'E). Pages 375-386 *in* J. Castellvi, editor. Actas del Segundo Symposium Espanol de Estudios Antarticos. |
|  | Woehler, E. J., G. W. Johnstone, and H. R. Burton. 1989a. The distribution and abundance of Adélie penguins, *Pygoscelis adeliae*, in the Mawson area and at the Rookery Islands (Specially Protected Area 2), 1981 and 1988. ANARE Research Notes 71, Australian Antarctic Division, Hobart. |
| Davis | Horne, R. S. C. 1983. The distribution of penguin breeding colonies on the Australian Antarctic Territory, Heard Island, the MacDonald Islands, and Macquarie Island. Australian Antarctic Division, Hobart. |
|  | Woehler, E. J., T. J. Tierney, and H. R. Burton. 1989b. The distribution and abundance of Adélie penguins, *Pygoscelis adeliae*, at the Vestfold Hills, 1973. ANARE Research Notes 70, Australian Antarctic Division. |
|  | Whitehead, M. D. and G. W. Johnstone. 1990. The distribution and estimated abundance of Adelie penguins breeding in Prydz Bay, Antarctica. Polar Biology **3**:91-98. |
| Casey | Orton, M. N. 1963. A brief survey of the fauna of the Windmill Islands, Wilkes Land, Antarctica. Emu **63**:15-22. |
|  | Horne, R. S. C. 1983. The distribution of penguin breeding colonies on the Australian Antarctic Territory, Heard Island, the MacDonald Islands, and Macquarie Island. Australian Antarctic Division, Hobart. |
|  | Woehler, E. J., D. J. Slip, L. M. Robertson, P. J. Fullagar, and H. R. Burton. 1991. The distribution, abundance and status of Adélie penguins *Pygoscelis adeliae* at the Windmill Islands, Wilkes Land, Antarctica. Marine Ornithology **19**:1-18. |
| Dumont  d’Urville | Prevost, J. 1963. Densités de peuplement et biomasses des vertébrés terrestres de l'archipel de Pointe Géologie, Terre Adélie. Terre et la Vie **1**:35-49. |
|  | Thomas, P. T. 1986. L'effectif des oiseaux nicheurs de l'archipel de Pointe Géologie (Terre Adélie) et son évolution au cours des trente dernières années. |
|  | Micol, T. and P. Jouventin. 2001. Long-term population trends in seven Antarctic seabirds at Pointe Géologie (Terre Adélie). Polar Biology **24**:175-185. |

**Table 3. Estimated population growth rate for five Adélie penguin regional populations, and all regional populations, over the last 30 years.**

| Region | Number of breeding sites  surveyed | Percent annual rate of change  (median, 95 percentile range) | |
| --- | --- | --- | --- |
| Syowa | 8 | 2.5 | 1.9 - 3.0 |
| Mawson | 14 | 2.4 | 1.4 – 4.5 |
| Davis | 47 | 2.1 | 1.5 - 2.6 |
| Casey | 11 | 1.9 | 1.4 – 2.5 |
| Dumont d’Urville | 19 | 1.8 | 1.6 - 1.9 |
| All regions | 99 | 1.9 | 1.3 - 2.4 |

**Table 4. Population estimates for sites surveyed at approximately decadal or greater frequency at each of five Adélie penguin regional populations.** Site locations are shown in Table 1. Estimates are totals for all sites in each region (Syowa 8 sites; Mawson: 13 sites; Davis: 21 sites; Casey: 11 sites; Dumont d’Urville: 19 sites).

| Region | Breeding season | Population estimate | |
| --- | --- | --- | --- |
|  |  | Median | 95% confidence interval |
| Syowa | 1975/76 | 815 | (754-890) |
|  | 1981/82 | 1,296 | (1,099-1,498) |
|  | 1982/83 | 1,420 | (1,291-1,553) |
|  | 1989/90 | 1,378 | (1,285-1,467) |
|  | 1991/92 | 1,391 | (1,276-1,491) |
|  | 1994/95 | 1,300 | (1,187-1,436) |
|  | 1995/96 | 1,504 | (1,352-1,676) |
|  | 1997/98 | 2,423 | (2,324-2,517) |
|  | 1998/99 | 2,036 | (1,839-2,152) |
|  | 1999/00 | 2,464 | (2,386-2,554) |
|  | 2000/01 | 2,101 | (1,885-2,417) |
|  | 2001/02 | 2,124 | (1,925-2,346) |
|  | 2002/03 | 3,072 | (2,902-3,227) |
|  | 2003/04 | 2,957 | (2,691-3,058) |
|  | 2004/05 | 3,325 | (3,181-3,491) |
|  | 2005/06 | 2,634 | (2,486-2,801) |
|  | 2006/07 | 3,047 | (2,931-3,170) |
|  | 2007/08 | 2,864 | (2,720-3,013) |
|  | 2008/09 | 2,255 | (2,136-2,371) |
|  | 2009/10 | 2,557 | (2,454-2,669) |
|  | 2010/11 | 2,047 | (1,981-2,117) |
| Mawson | 1973/74 | 55,881 | (51,046-62,090) |
|  | 1981/82 | 57,212 | (45,991-71,819) |
|  | 1988/89 | 72,427 | (66,312-125,039) |
|  | 2008/09 | 109,598 | (93,721-185,319) |
| Davis | 1973/74 | 186,258 | (169,210-204,240) |
|  | 1981/82 | 189,753 | (153,762-228,743) |
|  | 1993/94 | 225,252 | (204,745-247,203) |
|  | 2009/10 | 329,544 | (306,522-356,001) |
| Casey | 1961/62 | 22,455 | (20,385-24,614) |
|  | 1973/74 | 52,652 | (43,732-62,396) |
|  | 1989/90 | 54,700 | (52,790-57,145) |
|  | 2011/12 | 104,537 | (99,869-109,413) |
| Dumont d’Urville | 1960/61 | 20,067 | (17,797-24,471) |
|  | 1984/85 | 28,303 | (27,589-29,043) |
|  | 1999/00 | 47,643 | (46,243-49,171) |
|  | 2009/10 | 43,947 | (42,719-45,342) |
