# Supplementary material for: Spatially Extensive Standardized Surveys Reveal Widespread, Multi-Decadal Increase in East Antarctic Adélie Penguin Populations
Source: PLoS One. 2015 Oct 21;10(10):e0139877. doi: 10.1371/journal.pone.0139877 (PMC4619065; doi:10.1371/journal.pone.0139877)
Supplement: S2 File — (DOC) [file pone.0139877.s002.doc]

**1. Regional Population Habitat Areas**

Environmental covariates were measured in three primary habitat regions for each regional population:

*(1) On-land summer breeding areas*

The 99 breeding sites used in our analyses are clustered into closely-spaced groups of sites at each of the five regional populations located close to research stations (Figs 1 and B1).

*(2) At-sea summer foraging regions*

We defined a general summer foraging region extending northwards from each breeding regional population by 400 km and spanning 3º of longitude (Fig. B1). These regions aligned with Adélie penguin foraging ranges during the incubation and chick-rearing periods from foraging studies across East Antarctica (Kerry et al. 1997, Clarke et al. 2006, Kato et al. 2009, Wienecke et al. 2000, Cottin et al. 2012)

*(3) At-sea winter foraging regions*

We inferred a general winter foraging region for each regional population from the results of Clarke et al. (2003) for Mawson breeding penguins, as well as unpublished data on Adélie penguin winter foraging for populations breeding near Davis and Casey stations in East Antarctica. We assumed the pattern of winter foraging distribution would be similar for other regional populations across East Antarctica, and defined regions located largely westward of each regional population, spanning 30º of longitude, and extending north to 55ºS (Fig. B1).

**2. Regional population-scale environmental covariates**

*Air temperature and wind speed*

Air temperature and wind speed data for summer breeding areas were obtained from meteorological data recorded at nearby research stations and available from [www.antarctica.ac.uk/met/READER.surface/stationpt.html](http://www.antarctica.ac.uk/met/READER.surface/stationpt.html).

We sourced air temperature and wind speed data (m s^-1^ at 2 m and 10 m height, respectively) for the summer and winter foraging regions from the European Centre for Medium-range Weather Forecasting ERA-Interim reanalysis (Dee et al. 2011) (<http://data-portal.ecmwf.int/data/d/interim_daily/>).

Because foraging regions were large we used data averaged across multiple locations within the foraging ranges to represent meteorological conditions over the regions (two locations for summer foraging regions, eight locations for winter foraging regions, Fig. B1).

Data for summer breeding areas and summer foraging regions were averaged across December and January for each austral summer season. Temperatures for winter foraging regions were averaged across June, July and August each year.

*Sea-ice cover*

Sea-ice cover data for summer and winter foraging regions were obtained from satellite images from the National Snow and Ice Data Center (NSIDC). Data represent the total ice cover within the defined region with >15% sea ice concentration. Extraction code was provided by the Australian Antarctic Data Centre. Data for summer foraging regions were averaged over December and January for each austral summer season since satellite records began in 1980. Data for winter foraging areas were averaged over June, July and August each year.

*Sea-ice duration*

Sea-ice duration data were sourced from <http://www.cawcr.gov.au/staff/preid/seaice/japanese_study_new/sea_ice_duration_east_79_12>. We assumed that sea-ice duration data were most relevant as a covariate for summer foraging regions through their manifestation as ice-free areas or polynyas. Data were therefore averaged across grid cells within the summer foraging regions of each regional population.

*Southern Annular Mode (SAM)*

SAM data were obtained from the NOAA Climate Prediction Centre at <http://www.cpc.noaa.gov/products/precip/CWlink/daily_ao_index/aao/aao_index.html>. We calculated SAM values each year from 1980 for summer (December-January) and winter (June-August) periods.

**3. Local-scale environmental covariates**

*Area of potential habitat available for breeding*

We used the Southwell et al. (2009) coarse definition of potential breeding habitat as all ice-free land within 500 m of the ocean, and calculated the area available for breeding by subtracting the area currently occupied by a population of known size from the total area. To calculate the occupied area by an existing population we multiplied estimated population size in breeding pairs by an assumed density of 0.7 breeding pairs m^-2^ (Woehler and Riddle 1998).

**4. References**

Clarke, J., L. M. Emmerson, and P. Otahal. 2006. Environmental conditions and life history constraints determine foraging range in breeding Adélie penguins. Marine Ecology Progress Series **310**:247-261.

Clarke, J., K. Kerry, C. Fowler, R. Lawless, S. Eberhard, and R. Murphy. 2003. Post-fledging and winter migration of Adélie penguins *Pygoscelis adeliae* in the Mawson region of East Antarctica. Marine Ecology Progress Series **25**:717-720.

Cottin, M., B. Raymond, A. Kato, F. Amelineau, Y. Le Maho, T. Raclot, B. Galton-Fenzi, A. Meijers, and Y. Ropert-Coudert. 2012. Foraging strategies of male Adélie penguins during their first incubation trip in relation to environmental conditions. Marine Biology **159**:1843-1852.

Dee, D. P., S. M. Uppala, A. J. Simmons, P. Berrisford, P. Poli, S. Kobayashi, U. Andrae, M. A. Balmaseda, G. Balsamo, P. Bauer, P. Bechtold, A. C. M. Beljaars, L. van de Berg, J. Bidlot, N. Bormann, C. Delsol, R. Dragani, M. Fuentes, A. J. Geer, L. Haimberger, S. B. Healy, H. Hersbach, E. V. Holm, L. Isaksen, P. Kallberg, M. Kohler, M. Matricardi, A. P. McNally, B. M. Monge-Sanz, J.-J. Morcrette, B.-K. Park, C. Peubey, P. de Rosnay, C. Tavolato, J.-N. Thepaut, and F. Vitart. 2011. The ERA-Interim reanalysis: configuration and performance of the data assimilation system. Quarterly Journal of the Royal Meteorological Society **137**:553-597.

Kato, A., A. Yoshioka, and K. Sato. 2009. Foraging behavior of Adélie penguins during incubation period in Lutzow-Holm Bay. Polar Biology **32**:181-186.

Kerry, K. R., J. R. Clarke, S. Eberhard, H. Gardner, R. M. Lawless, R. Tremont, B. C. Wienecke, S. Corsolini, S. Focardi, E. Franchi, D. Rodary, and R. Thomson. 1997. The foraging range of Adélie penguins - implications for CEMP and interactions with the krill fishery. CCAMLR Science **4**:75-87.

Southwell, C., D. Smith, and A. Bender. 2009. Incomplete search effort: a potential source of bias in estimates of Adélie penguin breeding populations in the Australian Antarctic Territory. Polar Record **45**:1-6.

Wienecke, B. C., R. Lawless, D. Rodary, C.-A. Bost, R. Thomson, T. Pauly, G. Robertson, K. R. Kerry, and Y. LeMaho. 2000. Adélie penguin foraging behaviour and krill abundance along the Wilkes and Adelie land coasts, Antarctica. Deep Sea Research II **47**:2573-2587.

Woehler, E. J., and Riddle, M. 1998. Spatial relationships of Adélie penguin colonies: implications for assessing population changes from remote imagery. Antarctic Science **10**:449-454.

|  |  |  |  |  |
| --- | --- | --- | --- | --- |

**Figure 1. Time series (five-year moving average) of the Southern Annular Mode in summer, and air temperature and wind speed in the summer breeding areas of five Adélie penguin regional populations in East Antarctica. The horizontal bar graph at the bottom of the figure shows the multi-year periods over which population growth rates were estimated for the five regional populations. Regional populations are distinguished by colour: black: Syowa; red: Mawson; orange: Davis; green: Casey; blue: Dumont d’Urville.**

**Figure 2. Time series (five-year moving average) of the Southern Annular Mode in summer, and air temperature, wind speed, sea-ice cover and sea-ice duration in the summer foraging regions of five Adélie penguin regional populations in East Antarctica. The horizontal bar graph at the bottom of the figure shows the multi-year periods over which population growth rates were estimated for the five regional populations. Regional populations are distinguished by colour: black: Syowa; red: Mawson; orange: Davis; green: Casey; blue: Dumont d’Urville.**

**Figure 3. Time series (five-year moving average) of the Southern Annular Mode in winter, and air temperature, wind speed and sea-ice cover in the winter foraging regions of five Adélie penguin regional populations in East Antarctica. The horizontal bar graph at the bottom of the figure shows the multi-year periods over which population growth rates were estimated for the five regional populations. Regional populations are distinguished by colour: black: Syowa; red: Mawson; orange: Davis; green: Casey; blue: Dumont d’Urville.**

**Table 1. Multi-decadal trends for the Southern Annular Mode, and for air temperature, wind speed, sea-ice concentration and sea-ice duration for five Adélie penguin regional populations in East Antarctica during summer (December-January) and winter (June-August). Trends are indicated by the slopes of linear regressions of environmental covariates against year across the full time series. Slope units are: temperatures: ºC century^-1^; winds: m s^-1^ per century; sea-ice cover: km^2^ of ice per decade; sea-ice duration: days decade^-1^. *p*-values <0.01 in red.**

| **Habitat region** | **Environmental covariate** | **Regional population** | **Time period** | ***Slope*** | ***p*-value** |
| --- | --- | --- | --- | --- | --- |
| All regions | Southern Annular Mode | - | 1980-2013 | 0.03 | 0.082 |
| Summer breeding area | Air temperature | Syowa | 1960-1961, 1967-2013 | -0.10 | 0.890 |
|  |  | Mawson | 1955-2013 | -1.32 | 0.055 |
|  |  | Davis | 1958-1964,1970-2013 | 0.72 | 0.350 |
|  |  | Casey | 1960-2013 | -0.60 | 0.355 |
|  |  | Dumont d’Urville | 1957-2013 | 0.33 | 0.591 |
|  | Wind speed | Syowa | 1960-1962, 1967-2013 | 1.82 | 0.094 |
|  |  | Mawson | 1955-2013 | 2.24 | 0.015 |
|  |  | Davis | 1958-2013 | 3.26 | 0.000 |
|  |  | Casey | 1961-2013 | 2.10 | 0.008 |
|  |  | Dumont d’Urville | 1957-2013 | -3.79 | 0.000 |

Table 1 continued.

| **Habitat region** | **Environmental covariate** | **Regional population** | **Time period** | ***Slope*** | ***p*-value** |
| --- | --- | --- | --- | --- | --- |
| Summer foraging region | Air temperature | Syowa | 1980-2013 | -2.16 | 0.016 |
|  |  | Mawson | 1980-2013 | -2.39 | 0.067 |
|  |  | Davis | 1980-2013 | -1.68 | 0.109 |
|  |  | Casey | 1980-2013 | -3.84 | 0.000 |
|  |  | Dumont d’Urville | 1980-2013 | -0.76 | 0.498 |
|  | Wind speed | Syowa | 1980-2013 | 0.97 | 0.074 |
|  |  | Mawson | 1980-2013 | 0.37 | 0.428 |
|  |  | Davis | 1980-2013 | 0.80 | 0.064 |
|  |  | Casey | 1980-2013 | 0.32 | 0.571 |
|  |  | Dumont d’Urville | 1980-2013 | 0.35 | 0.655 |
|  | Sea-ice cover | Syowa | 1980-2013 | 110 | 0.924 |
|  |  | Mawson | 1980-2013 | 2,390 | 0.031 |
|  |  | Davis | 1980-2013 | 1,490 | 0.146 |
|  |  | Casey | 1980-2013 | 700 | 0.434 |
|  |  | Dumont d’Urville | 1980-2013 | 1,680 | 0.077 |
|  | Sea-ice duration | Syowa | 1979-2011 | 3.19 | 0.249 |
|  |  | Mawson | 1979-2011 | 6.67 | 0.009 |
|  |  | Davis | 1979-2011 | -0.79 | 0.800 |
|  |  | Casey | 1979-2011 | 0.58 | 0.846 |
|  |  | Dumont d’Urville | 1979-2011 | 3.49 | 0.253 |

**Table 1 continued.**

| **Habitat region** | **Environmental covariate** | **Regional population** | **Time period** | ***Slope*** | ***p*-value** |
| --- | --- | --- | --- | --- | --- |
| Winter foraging region | Southern Annular Mode | - | 1980-2013 | 0.00 | 0.934 |
|  | Air temperature | Syowa | 1980-2013 | -1.84 | 0.529 |
|  |  | Mawson | 1980-2013 | -0.56 | 0.794 |
|  |  | Davis | 1980-2013 | 1.88 | 0.334 |
|  |  | Casey | 1980-2013 | 0.55 | 0.783 |
|  |  | Dumont d’Urville | 1980-2013 | -3.25 | 0.055 |
|  | Wind speed | Syowa | 1980-2013 | 0.34 | 0.828 |
|  |  | Mawson | 1980-2013 | 0.46 | 0.177 |
|  |  | Davis | 1980-2013 | 0.91 | 0.047 |
|  |  | Casey | 1980-2013 | 0.49 | 0.230 |
|  |  | Dumont d’Urville | 1980-2013 | 0.02 | 0.972 |
|  | Sea-ice cover | Syowa | 1980-2013 | 49,100 | 0.069 |
|  |  | Mawson | 1980-2013 | 28,140 | 0.160 |
|  |  | Davis | 1980-2013 | 1,990 | 0.882 |
|  |  | Casey | 1980-2013 | -15,110 | 0.245 |
|  |  | Dumont d’Urville | 1980-2013 | 20,010 | 0.092 |

**Table 2. Analysis of variance results for decadal variation in air temperature, wind speed, sea-ice cover and sea-ice duration for five Adélie penguin regional populations in East Antarctica during summer (December-January) and winter (June-August). Decadal periods were: 1960s (1960-1969), 1970s (1970-1979), 1980s (1980-1989), 1990s (1990-1999) and 2000s (2000-2009). *p*-values <0.01 in red.**

| **Habitat region** | **Environmental covariate** | **Regional population** | **Time period** | ***df*** | ***F*** | ***p*-value** |
| --- | --- | --- | --- | --- | --- | --- |
| All regions | Southern Annular Mode | - | 1980s-2000s | 2,27 | 1.302 | 0.288 |
| Summer breeding area | Air temperature | Syowa | 1970s-2000s | 3,36 | 0.316 | 0.815 |
|  |  | Mawson | 1960s-2000s | 4,45 | 2.426 | 0.062 |
|  |  | Davis | 1970s-2000s | 3,36 | 1.073 | 0.373 |
|  |  | Casey | 1960s-2000s | 4,45 | 1.549 | 0.204 |
|  |  | Dumont d’Urville | 1960s-2000s | 4,45 | 1.714 | 0.164 |
|  | Wind speed | Syowa | 1970s-2000s | 4,41 | 7.163 | 0.000 |
|  |  | Mawson | 1960s-2000s | 4,45 | 4.412 | 0.004 |
|  |  | Davis | 1960s-2000s | 4,44 | 17.860 | 0.000 |
|  |  | Casey | 1960s-2000s | 4,44 | 3.846 | 0.009 |
|  |  | Dumont d’Urville | 1960s-2000s | 4,45 | 6.527 | 0.000 |

**Table 2 continued.**

| **Habitat region** | **Environmental covariate** | **Regional population** | **Time period** | ***df*** | ***F*** | ***p*-value** |
| --- | --- | --- | --- | --- | --- | --- |
| Summer foraging region | Air temperature | Syowa | 1980s-2000s | 2,27 | 2.028 | 0.151 |
|  |  | Mawson | 1980s-2000s | 2,27 | 0.744 | 0.486 |
|  |  | Davis | 1980s-2000s | 2,27 | 0.524 | 0.598 |
|  |  | Casey | 1980s-2000s | 2,27 | 9.426 | 0.001 |
|  |  | Dumont d’Urville | 1980s-2000s | 2,27 | 0.129 | 0.879 |
|  | Wind speed | Syowa | 1980s-2000s | 2,27 | 0.932 | 0.406 |
|  |  | Mawson | 1980s-2000s | 2,27 | 0.097 | 0.908 |
|  |  | Davis | 1980s-2000s | 2,27 | 2.219 | 0.128 |
|  |  | Casey | 1980s-2000s | 2,27 | 1.265 | 0.298 |
|  |  | Dumont d’Urville | 1980s-2000s | 2,27 | 0.414 | 0.665 |
|  | Sea-ice cover | Syowa | 1980s-2000s | 2,27 | 1.238 | 0.306 |
|  |  | Mawson | 1980s-2000s | 2,27 | 1.247 | 0.303 |
|  |  | Davis | 1980s-2000s | 2,27 | 2.952 | 0.069 |
|  |  | Casey | 1980s-2000s | 2,27 | 0.645 | 0.533 |
|  |  | Dumont d’Urville | 1980s-2000s | 2,27 | 1.529 | 0.235 |
|  | Sea-ice duration | Syowa | 1980s-2000s | 2,27 | 0.179 | 0.837 |
|  |  | Mawson | 1980s-2000s | 2,27 | 0.548 | 0.585 |
|  |  | Davis | 1980s-2000s | 2,27 | 0.931 | 0.407 |
|  |  | Casey | 1980s-2000s | 2,27 | 3.031 | 0.065 |
|  |  | Dumont d’Urville | 1980s-2000s | 2,27 | 1.561 | 0.228 |

**Table 2 continued.**

| **Habitat region** | **Environmental covariate** | **Regional population** | **Time period** | ***df*** | ***F*** | ***p*-value** |
| --- | --- | --- | --- | --- | --- | --- |
| Winter foraging region | Southern Annular Mode | - | 1980s-2000s | 2,27 | 0.238 | 0.790 |
|  | Air temperature | Syowa | 1980s-2000s | 2,27 | 0.038 | 0.963 |
|  |  | Mawson | 1980s-2000s | 2,27 | 0.169 | 0.845 |
|  |  | Davis | 1980s-2000s | 2,27 | 3.917 | 0.032 |
|  |  | Casey | 1980s-2000s | 2,27 | 0.629 | 0.540 |
|  |  | Dumont d’Urville | 1980s-2000s | 2,27 | 0.838 | 0.443 |
|  | Wind speed | Syowa | 1980s-2000s | 2,27 | 0.533 | 0.593 |
|  |  | Mawson | 1980s-2000s | 2,27 | 1.047 | 0.364 |
|  |  | Davis | 1980s-2000s | 2,27 | 0.491 | 0.617 |
|  |  | Casey | 1980s-2000s | 2,27 | 0.116 | 0.891 |
|  |  | Dumont d’Urville | 1980s-2000s | 2,27 | 0.232 | 0.794 |
|  | Sea-ice cover | Syowa | 1980s-2000s | 2,27 | 1.010 | 0.378 |
|  |  | Mawson | 1980s-2000s | 2,27 | 0.188 | 0.830 |
|  |  | Davis | 1980s-2000s | 2,27 | 1.397 | 0.265 |
|  |  | Casey | 1980s-2000s | 2,27 | 0.461 | 0.636 |
|  |  | Dumont d’Urville | 1980s-2000s | 2,27 | 0.6767 | 0.517 |

**Table 3. Results of pair-wise Pearson product-moment correlation tests (*r* and *p*-values) between trend in environmental covariates over the multi-year periods used to estimate regional population change. S.SAM: Southern Annular Mode in summer; W.SAM: Southern Annular Mode in winter; SBA: summer breeding area; SFR: summer foraging region; WFR: winter foraging region; AT: air temperature, WS: wind speed; SIC: sea-ice cover; SID: sea-ice duration. *p*-values <0.01 in red.**

|  | S.SAM | W.SAM | SBA.AT | SBA.WS | SFR.AT | SFR.WS | SFR.SIC | SFR.SID | WFR.AT | WFR.WS | WFR.SIC |
| --- | --- | --- | --- | --- | --- | --- | --- | --- | --- | --- | --- |
| S.SAM | - | - | - | - | - | - | - | - | - | - | - |
| W.SAM | -0.368  0.266 | - | - | - | - | - | - | - | - | - | - |
| SBA.AT | -0.520  0.101 | 0.247  0.463 | - | - | - | - | - | - | - | - | - |
| SBA.WS | -0.063  0.853 | -0.427  0.190 | -0.081  0.765 | - | - | - | - | - | - | - | - |
| SFR.AT | -0.390  0.265 | -0.264  0.461 | 0.463  0.178 | -0.349  0.323 | - | - | - | - | - | - | - |
| SFR.WS | 0.699  0.024 | -0.173  0.633 | -0.361  0.306 | 0.338  0.339 | -0.728  0.017 | - | - | - | - | - | - |
| SFR.SIC | 0.319  0.338 | -0.429  0.187 | 0.066  0.847 | -0.227  0.501 | 0.569  0.086 | -0.006  0.987 | - | - | - | - | - |
| SFR.SID | 0.437  0.179 | 0.273  0.417 | -0.203  0.549 | -0.197  0.561 | -0.542  0.105 | 0.624  0.053 | 0.150  0.658 | - | - | - | - |
| WFR.AT | -0.605  0.064 | 0.238  0.509 | 0.022  0.953 | 0.189  0.601 | 0.019  0.956 | -0.537  0.109 | -0.652  0.041 | -0.596  0.069 | - | - | - |
| WFR.WS | -0.354  0.315 | 0.393  0.261 | -0.464  0.176 | 0.313  0.378 | -0.357  0.311 | -0.208  0.565 | -0.719  0.019 | 0.000  0.999 | 0.562  0.092 | - | - |
| WFR.SIC | 0.719  0.013 | -0.121  0.723 | -0.192  0.571 | -0.244  0.469 | -0.337  0.340 | 0.649  0.042 | 0.439  0.176 | 0.858  0.001 | -0.828  0.003 | -0.342  0.333 | - |
