# Supplementary material for: Spatially Extensive Standardized Surveys Reveal Widespread, Multi-Decadal Increase in East Antarctic Adélie Penguin Populations
Source: PLoS One. 2015 Oct 21;10(10):e0139877. doi: 10.1371/journal.pone.0139877 (PMC4619065; doi:10.1371/journal.pone.0139877)
Supplement: S3 File — (DOC) [file pone.0139877.s003.doc]

**Table 1. Pearson’s product-moment correlations (*r*) between Adélie penguin regional population growth rates and trends in environmental covariates with no lag and with a five-year lag. *p*-values <0.01 in red.**

| **Time lag** | **Habitat region** | **Environmental covariate** | ***df*** | ***r*** | ***p*-value** |
| --- | --- | --- | --- | --- | --- |
| None | All regions | Southern Annular Mode | 9 | -0.442 | 0.174 |
|  | Summer breeding area | Air temperature | 14 | 0.111 | 0.684 |
|  |  | Wind speed | 14 | 0.471 | 0.065 |
|  | Summer foraging region | Air temperature | 8 | 0.332 | 0.348 |
|  |  | Wind speed | 8 | -0.616 | 0.058 |
|  |  | Sea-ice cover | 9 | -0.055 | 0.873 |
|  |  | Sea-ice duration | 9 | -0.292 | 0.384 |
|  | Winter foraging region | Southern Annular Mode | 9 | 0.020 | 0.953 |
|  |  | Air temperature | 8 | 0.476 | 0.164 |
|  |  | Wind speed | 8 | 0.685 | 0.029 |
|  |  | Sea-ice cover | 9 | -0.454 | 0.161 |

**Table 1 (continued)**

| **Time lag** | **Habitat region** | **Environmental covariate** | ***df*** | ***r*** | ***p*-value** |
| --- | --- | --- | --- | --- | --- |
| 5 years | All regions | Southern Annular Mode | 9 | 0.599 | 0.051 |
|  | Summer breeding area | Air temperature | 14 | -0.638 | 0.008 |
|  |  | Wind speed | 14 | 0.142 | 0.599 |
|  | Summer foraging region | Air temperature | 7 | -0.694 | 0.038 |
|  |  | Wind speed | 7 | 0.447 | 0.228 |
|  |  | Sea-ice cover | 9 | -0.125 | 0.715 |
|  |  | Sea-ice duration | 9 | 0.029 | 0.933 |
|  | Winter foraging region | Southern Annular Mode | 9 | 0.564 | 0.071 |
|  |  | Air temperature | 7 | 0.845 | 0.004 |
|  |  | Wind speed | 7 | 0.632 | 0.068 |
|  |  | Sea-ice cover | 9 | -0.753 | 0.008 |
