# Supplementary material for: Correction: Spatially Extensive Standardized Surveys Reveal Widespread, Multi-Decadal Increase in East Antarctic Adélie Penguin Populations
Source: PLoS One. 2016 Oct 28;11(10):e0165989. doi: 10.1371/journal.pone.0165989 (PMC5085017; doi:10.1371/journal.pone.0165989)
Supplement: S1 File — (PDF) [file pone.0165989.s001.pdf]

RESEARCH ARTICLE

Spatially Extensive Standardized Surveys Reveal Widespread, Multi-Decadal Increase in East Antarctic Adélie Penguin Populations

Colin Southwell[1]*, Louise Emmerson[1], John McKinlay[1], Kym Newbery[1], Akinori Takahashi[2], Akiko Kato[2¤], Christophe Barbraud[3], Karine DeLord[3], Henri Weimerskirch[3]

1 Australian Antarctic Division, Department of the Environment, Kingston, Tasmania, Australia, **2** National Institute of Polar Research, Tachikawa, Tokyo, Japan, **3** Centre d'Etudes Biologiques de Chizé, UMR7372 CNRS / Univ La Rochelle, Villiers en Bois, France

¤ Current address: Universite de Strasbourg, IPHC, Strasbourg, France
* colin.southwell@aad.gov.au

OPEN ACCESS

Citation: Southwell C, Emmerson L, McKinlay J, Newbery K, Takahashi A, Kato A, et al. (2015) Spatially Extensive Standardized Surveys Reveal Widespread, Multi-Decadal Increase in East Antarctic Adélie Penguin Populations. PLoS ONE 10(10): e0139877. doi:10.1371/journal.pone.0139877

Editor: Sébastien Descamps, Norwegian Polar Institute, NORWAY

Received: April 20, 2015

Accepted: September 17, 2015

Published: October 21, 2015

Copyright: © 2015 Southwell et al. This is an open access article distributed under the terms of the Creative Commons Attribution License, which permits unrestricted use, distribution, and reproduction in any medium, provided the original author and source are credited.

Data Availability Statement: All relevant population data are within the paper and its Supporting Information files.

Funding: The work was carried out under funding to (i) Australian Antarctic Science projects 2722, 4087 and 4088, (ii) Japanese Antarctic Research Expeditions programs AMB1 and AP12, and (iii) Institut Paul Emile Victor (IPEV), Zone Atelier Antarctique (CNRS-INEE), and Terres Australes et Antarctiques Françaises. The funders had no role in study design, data collection and analysis, decision to publish, or preparation of the manuscript.

Abstract

Seabirds are considered to be useful and practical indicators of the state of marine ecosystems because they integrate across changes in the lower trophic levels and the physical environment. Signals from this key group of species can indicate broad scale impacts or response to environmental change. Recent studies of penguin populations, the most commonly abundant Antarctic seabirds in the west Antarctic Peninsula and western Ross Sea, have demonstrated that physical changes in Antarctic marine environments have profound effects on biota at high trophic levels. Large populations of the circumpolar-breeding Adélie penguin occur in East Antarctica, but direct, standardized population data across much of this vast coastline have been more limited than in other Antarctic regions. We combine extensive new population survey data, new population estimation methods, and re-interpreted historical survey data to assess decadal-scale change in East Antarctic Adélie penguin breeding populations. We show that, in contrast to the west Antarctic Peninsula and western Ross Sea where breeding populations have decreased or shown variable trends over the last 30 years, East Antarctic regional populations have almost doubled in abundance since the 1980's and have been increasing since the earliest counts in the 1960's. The population changes are associated with five-year lagged changes in the physical environment, suggesting that the changing environment impacts primarily on the pre-breeding age classes. East Antarctic marine ecosystems have been subject to a number of changes over the last 50 years which may have influenced Adélie penguin population growth, including decadal-scale climate variation, an inferred mid-20th century sea-ice contraction, and early-to-mid 20th century exploitation of fish and whale populations.

Competing Interests: The authors have declared that no competing interests exist.

Introduction

Antarctic environments have been changing at different rates and in different ways around the continent. Decadal-scale shifts in atmospheric circulation through the Southern Annular Mode (SAM) have influenced wind speed and temperature differentially from region to region. In the most dramatic example of this, a predominantly positive SAM in recent decades has resulted in warm, north-to-south, largely cross-shore winds in the western Antarctic Peninsula (WAP) and cold, south-to-north, off-shore winds in the Ross Sea [1,2]. Satellite observations have revealed widespread and spatially homogeneous, but opposing, changes in sea-ice cover and duration associated with these varying atmospheric conditions in the two regions. In the WAP the sea-ice season has shortened by 30–40 days decade^{-1} and sea-ice extent has decreased by 0.5–0.7% decade^{-1}, whereas in the Ross Sea the sea-ice season has lengthened by 20–30 days decade^{-1} and sea-ice extent has increased by 0.3–0.5% decade^{-1} [1,3,4]. Such changes in the physical environment are expected to have cascading effects through the biological system to the higher-order predators because of the strong links between physical features such as sea-ice and the lower trophic levels of the food web [5–10].

For higher-order predators, the impacts of a changing physical environment can be both direct and indirect. For example, extensive fast-ice can directly reduce access to marine prey by forming a physical barrier to the ocean for Adélie penguins [11,12], and climate variation has been proposed to indirectly impact Antarctic fur seal pup production through its effect on krill [13,14]. Consequently, higher-order predators are often considered as good indicators of ecosystem change because they integrate over the entire trophic web and are susceptible to changes in the physical environment.

As an abundant higher-order marine predator with a circumpolar breeding distribution, the Adélie penguin (*Pygoscelis adeliae*) is subject to the full range of environmental change and variability around the continent. Recent syntheses of long-term, regional-scale, direct surveys of Adélie penguin populations in the WAP and western Ross Sea (WRS) regions [15,16] have provided important insights into biological change and ecological processes in these regions of contrasting environmental change. Adélie penguin populations have decreased at most sites in the northern and central WAP but increased at southerly sites [15]. Although the decreases have been associated with reduced sea-ice and krill biomass along the WAP [15,17], ecosystem modelling suggests that krill biomass should be sufficient to support the Adélie populations and that factors other than reduced krill biomass may be regulating Adélie population decreases in the WAP [18]. In the WRS Adélie population trends are thought to be related to climate variation and the effects of whaling and fishing [16]. The spatially extensive surveys by [15] found significant local-scale variation in population change within a context of broader regional change, promoting caution against extrapolating to regional-scale dynamics and mechanisms from a single or few populations.

In comparison to the WAP and the Ross Sea, changes in the physical environment in East Antarctica (EA) appear to be less pronounced, and little is known about changes in lower trophic levels of the biological system. Decadal-scale changes in sea-ice extent and duration in EA have been mixed and characterized by pockets of locally strong positive and negative trends set in a broader background of weaker change [1,3,19]. In contrast to a strong decline in krill abundance in the WAP, the patterns of decadal-scale change of krill abundance in EA are variable where limited data exist [20]. Against the background of weaker and more variable environmental change than in the WAP and Ross Sea regions, an assessment of decadal-scale change of Adélie penguin populations across EA is an important contribution to Antarctic and Southern Ocean ecology that could lead to improved understanding of broader ecosystem processes and change around the continent.

The regional-scale assessments of Adélie penguin population change in the WAP and WRS have been facilitated by standardized collection or analysis of direct population count data, ensuring that estimates of the direction and rate of population change are accurate and reliable. In the WRS, direct counts from aerial photographic surveys have been undertaken at the optimal time of the breeding season for population estimation (early December) for several decades [16,21,22]. Surveying at this time, when only males incubating eggs are present at breeding sites, ensures that the count closely represents the standard metric for population estimation (the number of breeding pairs during incubation when few nests have failed). In the WAP, direct ground counts have been conducted at varying (often sub-optimal) times within the breeding season over several decades, but the counts have been standardized to the optimal time using off-peak census correction factors [15,23] to ensure the rate of population change was estimated reliably.

To match the quality and extent of Adélie penguin population assessments in the WAP and WRS, a complimentary assessment in EA requires standardized, spatially extensive population count data to ensure comparisons over time are robust to methodological variation and accurately represent regional-scale change. A number of studies have analysed long-term direct count data from regional populations in EA between longitudes 40°-140°E [24–30]. In the most spatially extensive analysis [30], data were available for multiple breeding sites in two regions (Pt Géologie and Syowa at the eastern and western ends of EA respectively), but data were analysed for only two small breeding populations along the extensive coastline between these regions (hereafter referred to as 'central EA' between longitudes 50°-120°E) where ~150 breeding sites (or ~70% of all EA breeding sites) exist. The spatial extent of this assessment was therefore sparse for most of the EA population. A more spatially extensive study by [31] estimated the direction of population change by comparing published historical population estimates with recent estimates derived from satellite imagery. While these studies have been important contributions, they relied on published historical Adélie penguin population estimates as a baseline to estimate change. A recent study has demonstrated that many of these published population estimates in central EA have biases and uncertainties that have not previously been recognized, and that conclusions about Adélie penguin population change are improved when re-constructed estimates of historical abundance are used as a baseline instead of published estimates [32]. Thus, until this study, a standardized, spatially extensive assessment of Adélie penguin population change has not been achieved for this large region of Antarctica.

In this paper we address this gap by presenting the most comprehensive assessment to date of Adélie penguin population change across EA. Our assessment is based on a comparison of recent spatially extensive direct population surveys and re-constructed historical survey data from multiple breeding sites and regions across EA. An important feature of our work is the use of new standardization methods and data to ensure population estimates derived from recent and historical counts can be reliably compared and estimated rates of change are unbiased. We synthesize these standardized data to estimate spatio-temporal variation in the rate of population change across a continuum of spatial scales from the total EA population through to regional and local populations. We also examine environmental change in regions used by the penguins for breeding and foraging to investigate potential drivers or constraints of population change. Our results fill a large and important missing piece of the circum-Antarctic picture of this key biological indicator species.

Materials and Methods

Population counts

Our assessment was based on direct counts of Adélie penguins breeding at sites located along 4,500 km of the EA coastline between 40°E–140°E (Fig 1). We use the term 'breeding site' to

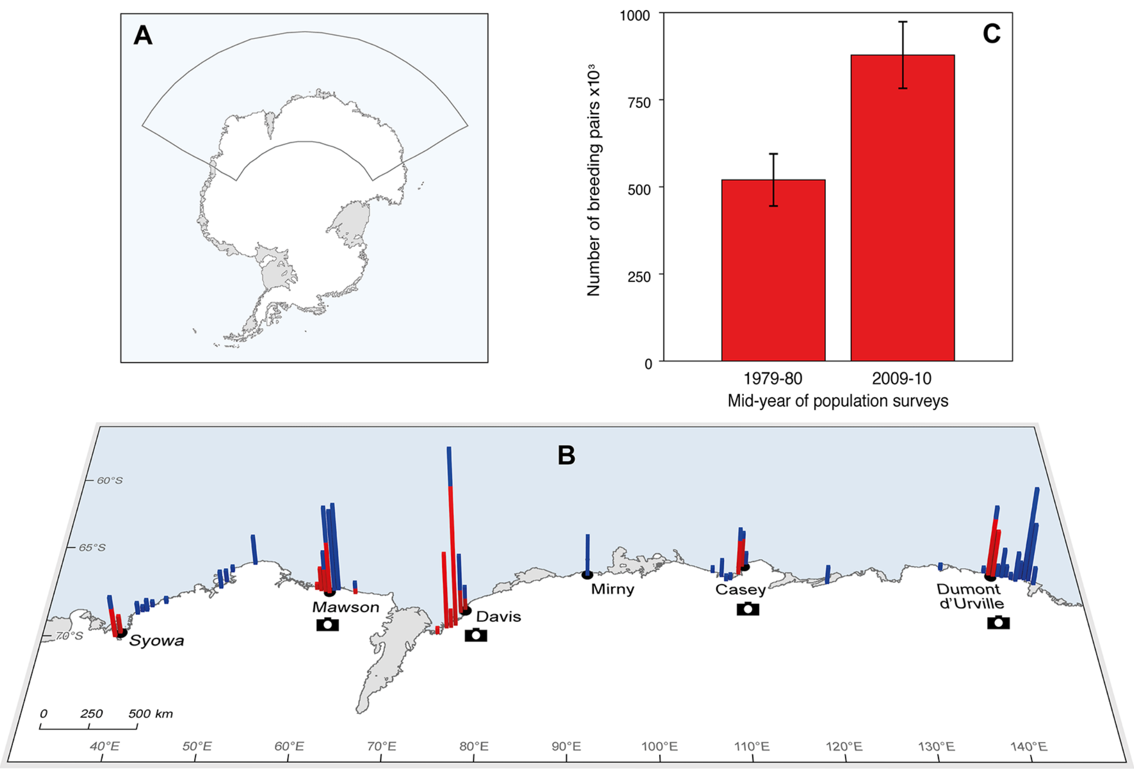

Fig 1. Change in the East Antarctic Adélie penguin breeding population over the last 30 years. (A) Extent of survey region, (B) distribution of Adélie penguin breeding sites, population counts and cameras within the survey region, and (C) estimated Adélie penguin breeding population at 99 sites surveyed recently and 30 years ago. In panel B, bars indicate the number of sites within half-degree longitude increments, with red bars for repeat surveys that contributed to estimates of population change and blue bars for sites that did not contribute to estimates of change. For scale, the largest incremental bar indicates 30 breeding sites. The maps were created using GIS data from the SCAR Antarctic Digital Database.

doi:10.1371/journal.pone.0139877.g001

refer to a distinct geographical feature such as an island or outcrop of continental rock where Adélie penguins are known to breed. There are >200 Adélie penguin breeding sites along the EA coast in several spatially-distinct clusters separated by coastline devoid of breeding habitat due to the presence of ice-cliffs and floating ice-shelves (Fig 1). We refer to breeding populations at the spatially-distinct clusters of breeding sites as 'regional populations', and at the individual breeding sites as 'local populations'.

To estimate long-term population change over multiple spatial scales in EA, we conducted direct population counts at 99 breeding sites across five regional populations (S1 File) where counts had been made three or more decades ago (Fig 1). The vast region, varied logistic support, variable size of populations, and changing weather and sea-ice conditions meant that methods for recent and historical direct population counts varied from site to site. Sites close to research stations were usually accessed and counted from the ground. More distant sites were accessed by aircraft and counts were made from overlapping aerial photographs taken with a fixed, vertical-facing camera or from aerial photographs taken with a hand-held, obliquely-facing camera. Ground-based observers counted occupied nests at small populations but sometimes switched to counting adults at larger populations if they were not confident of reliably observing nests from their oblique view. Occupied nests are difficult to identify in aerial photographs and consequently all aerial-based counts were of adults. Small populations were counted entirely (i.e. census counts), but this was not practical for large populations where sample counts were made instead. It was often not possible to obtain direct counts at the

optimal time for population estimation (see below) because of weather and logistic constraints. Historical counts were generally less optimally-timed than recent counts. The dates of counts varied from early-November to late-January.

Field work was conducted in accordance with permits issued by (i) the Australian Antarctic Ethics Committee for Australian Antarctic Science projects 2722, 4087 and 4088, (ii) the Committee on Bioscience Program at the National Institute of Polar Research for Japanese Antarctic Research Expeditions programs AMB1 and AP12, and (iii) Terres Australes et Antarctiques Françaises authorities for IPEV program 109 under the Institut Paul Emile Victor, Zone Atelier de Recherches sur l'Environnement Antarctique et Subantarctique (CNRS-INEE), and Terres Australes et Antarctiques Françaises. All field procedures were specifically approved as part of obtaining the permits. The field studies did not involve endangered or protected species.

Standardizing population count data

Rigorous standardization of count data is a critical feature of our study. Standardization was important because the counting methods and dates varied considerably in both the recent and historical surveys. The standardization methods were developed to account for detection and availability biases and their uncertainties that are pertinent to colonial-breeding species. We standardized or adjusted raw counts to an estimate of the maximum number of occupied nests established early in the incubation period before nests have failed (late-November to early-December). The standardization process applied non-parametric bootstrap methods [33–35] to raw count data and adjustment data. The raw count data comprised the population unit counted (adults, nests or chicks), the date of the count, and the count value. Adjustment data specific to the count date and population unit counted were obtained from a network of remotely-operating cameras across EA (Fig 1) [36]. Instead of using historical population estimates from the published literature as in previous EA assessments, we de-constructed the historical estimates to the raw count data and adjusted the raw counts using camera-derived adjustment data [32] in the same way as for recent counts. This ensured the same standardization process was applied to both the recent and historical count data.

Estimating population growth rate

We estimated annual population growth rate (*pgr*) over various spatial (total, regional, local) and temporal (multi-decadal, decadal) scales by fitting a linear regression of the natural logarithm of standardized population estimates against year and taking the slope as an estimate of *pgr* across the span of the time series. The growth estimates are also expressed as annual percentage growth rates. We also used generalized additive models (GAMs) to assess evidence for non-linear change over time at local sites with the richest time series in each region.

Environmental covariates

Population growth may be related to changes in the conditions prevailing at breeding areas, in summer foraging regions, and in winter foraging regions. Given the large scale of our study and the wide-ranging movements of Adélie penguins when foraging, there are many potential environmental covariates that could influence the penguins. We used a set of physical covariates that may directly or indirectly influence Adélie penguin population growth, and have been measured annually over at least the last three decades at spatial scales matching the breeding areas and foraging regions (air temperature and wind speed in all regions, sea-ice cover in summer and winter foraging regions, sea-ice duration in the summer foraging region, and the Southern Annular Mode (SAM) as a global atmospheric-climate index across all regions), to explore associations between environmental change and population change. These covariates

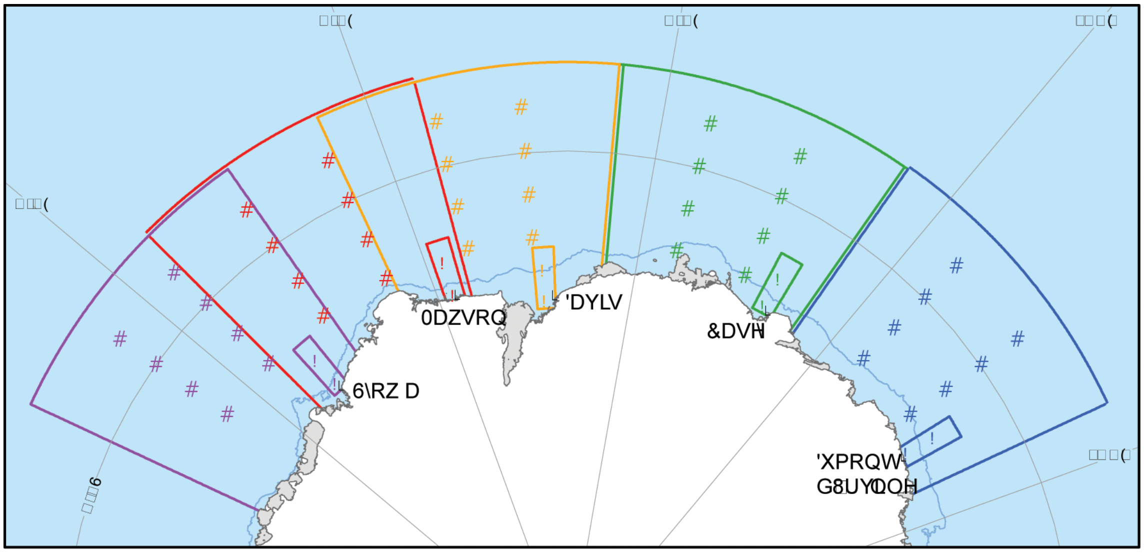

Fig 2. Boundaries of foraging regions for five regional Adélie penguin populations, and locations where environmental data were measured. Triangles: winter foraging regions; circles: summer foraging regions; squares: summer breeding areas. Purple: Syowa; red: Mawson; orange: Davis; green: Casey; blue: Dumont d'Urville. The map was created using GIS data from the SCAR Antarctic Digital Database.

doi:10.1371/journal.pone.0139877.g002

have been used in a number of similar analyses at smaller spatial scales [22–29]. The spatial and temporal extent of the covariates was based on known space usage for summer and winter breeding and foraging activities and was focussed on both summer and winter processes (Fig 2 and S2 File). We could not investigate associations between population growth and change in biological covariates such as prey (krill and fish) abundance, or potential proxies to prey abundance such as phytoplankton, because such biological time series do not exist over the required large spatial and temporal scales.

Environmental change

We assessed multi-decadal trends and decadal-scale variation in environmental covariates. To assess multi-decadal trends, we fitted a linear regression to each covariate time series against year, calculated the slope of the regression to quantify the direction and rate of change, and tested whether the slope differed from zero using a *t*-test. To assess decadal variation, we performed an analysis of variance on each covariate partitioned into decadal intervals. We tested for temporal auto-correlation in the environmental covariate time series and found no significant correlation with lags of 1–5 years in 42 of the 45 time series.

Associations between regional population growth and environmental change

We explored the associations between regional population growth rate and trends in environmental conditions. Analysis of these spatially extensive data was constrained by the low temporal frequency of historical population data for most regional populations (approximately decadal for Mawson, Davis, Casey and Dumont d'Urville) and a lack of consistency in the timing of historical population data between regional populations. Given these unavoidable limitations associated with retrospective analysis of historical data, we examined associations between population growth rates and environmental trends over multi-year periods defined by the available population data (three periods between successive points in abundance time series

for the Mawson, Davis, Casey and Dumont d'Urville regional populations; four periods defined by apparent break-points in more frequent data at Syowa (1975/76-1981/82, 1981/82-1994/95, 1994/95-2002/03, 2002/03-2009/10), S2 File). There was a further constraint in that while we could estimate population growth for the resulting 16 regional population/multi-year periods, we could not estimate environmental trend for the covariates in all the periods because satellite-derived environmental data were not available before 1977 (S2 File). Hence we used Pearsons' product-moment correlations between estimates of population growth and environmental trend over the multi-year periods instead of more sophisticated modelling approaches which require a more balanced matching between response and explanatory data. We also examined correlations shifted with a five-year time lag to assess potential lag between environmental change and population response related to recruitment of young into the breeding population [22,29]. Because we conducted multiple analyses of change and association, we set a stricter threshold for statistical significance at a level of 0.01 to reduce the chance of Type I errors.

Results

Population growth

Our estimate of the current breeding population at the 99 sites across EA is 69% (95% CI 44%-94%) higher than a re-constructed estimate for the same sites when surveyed 30 years ago (878,308 (807,070–974,025) pairs vs 520,050 (467,500–595,003) pairs). The difference is well beyond the uncertainties in the two estimates (Fig 1) and equates to a population growth rate estimate of pgr = 0.019 (0.013–0.024) or a percentage average rate of increase of 1.9% (1.3%-2.4%) yr^{-1} over the 30 years. All five regional populations have increased over the last 30 years, with growth rates ranging from 1.8% to 2.5% yr^{-1} (S1 File). However, there was some spatial heterogeneity in the growth rate of local populations within each regional population. Thus while the majority (84 of 99) of local populations increased, there were decreases at 15 local populations across four of the five regional populations (Fig 3). Overall, growth rates for local populations over the last 30 years ranged from -5.8% to 11.4% yr^{-1}.

Examination of data from a subset of 72 sites that had been surveyed at least four times in the last 50 years (Fig 4, S1 File) reveals decadal-scale variation within the long-term increases. The four regional populations with population data from the 1970s and 1980s showed an apparent flattening in the overall increasing trend during these decades, but the low temporal resolution of data makes it difficult to determine when these flat periods began and ended at each region. The overall increasing trend has eased in the last decade at the Syowa and Dumont d'Urville regional populations, with populations declining or remaining steady over this period. Data for the other three regional populations lacked the temporal resolution required to assess whether any similar easing of population growth has occurred in these regions in the last decade. Population growth rates over multi-year periods between time series points for the five regional populations ranged from -3.4% to 8.1% yr^{-1} (Fig 4).

Fitting GAMs to population count time series for the site with the richest time series in each regional population showed that populations at these sites increased at similar rates for 3–4 decades from the time of the first reported counts 50–60 years ago, but the rates of change have become more variable in the last 10–15 years as some populations continue to increase and others plateau or decline (Fig 5).

Environmental change

The multi-decadal and decadal trend and variation analyses of environmental covariates in the breeding areas and foraging regions indicated a clear change in wind speed (S2 File). Summer

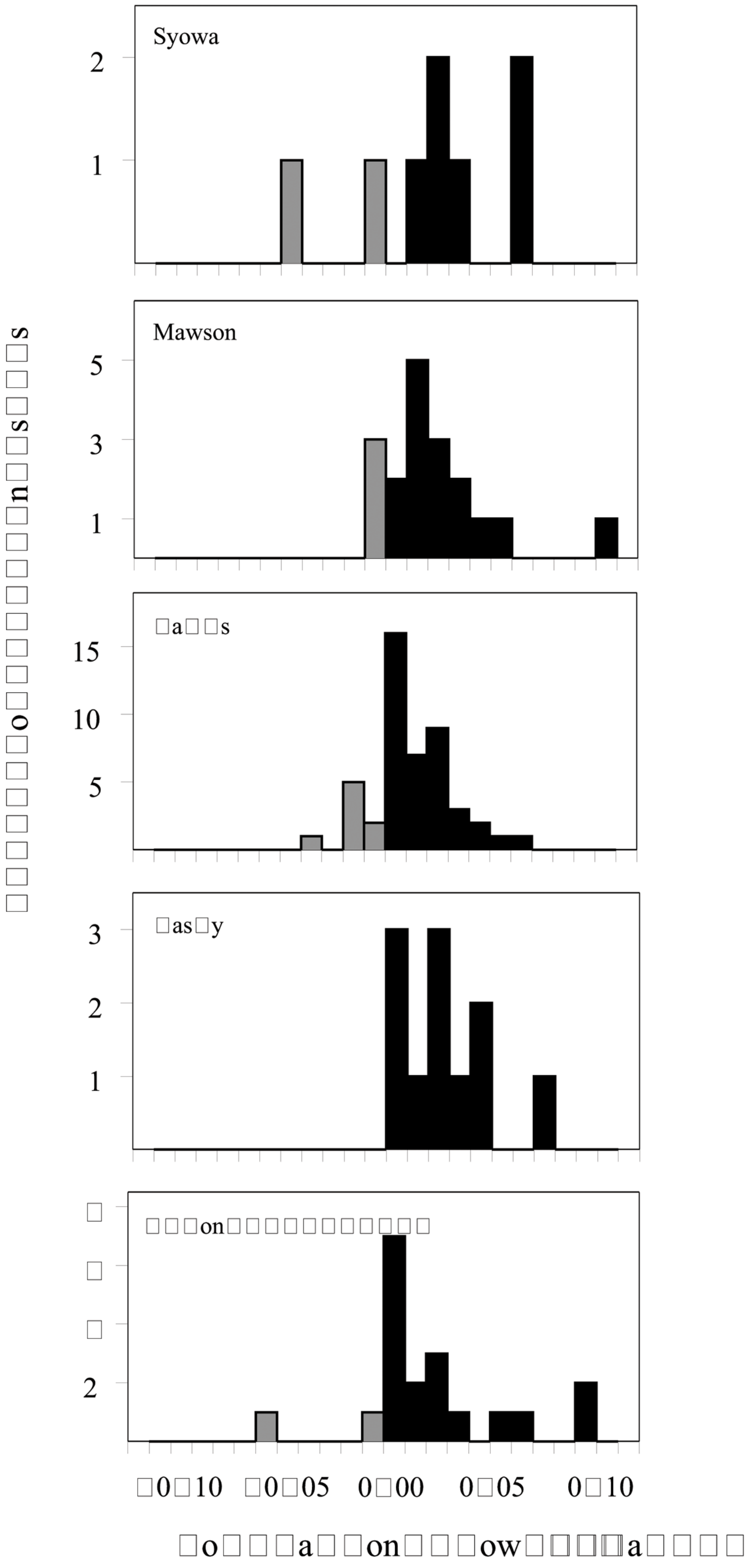
Syowa
Mawson
2
1
5
3
1
15
10
5
3
2
1
2

Fig 3. Distribution of local population growth rate within five regional populations across East Antarctica over the past 30 years. Black bars indicate positive population growth and grey bars negative growth.

doi:10.1371/journal.pone.0139877.g003

winds have strengthened at rates of 2–3 m s^{-1} per decade at the Syowa, Mawson, Davis and Casey breeding areas over the last 50–60 years, but have weakened at 4 m s^{-1} per decade at Dumont d'Urville (S2 File). Most of these changes were significant at the 0.01 level. Wind speed also showed a consistent strengthening trend in the summer and winter foraging regions of all regional populations, but no changes were statistically significant (S2 File). In contrast, trends in air temperatures in each habitat region were relatively weak and variable across regional populations. There was, however, clear synchrony across regional populations in the temporal pattern of summer air temperatures at the breeding areas, with high temperatures in the 1970s, 1980s and 2000s and low temperatures in the 1960s and 1990s (S2 File). Sea-ice cover showed an increasing but non-significant trend in all summer foraging regions and in all but the Casey winter foraging region, where there was a non-significant decreasing trend (S2 File). Sea-ice duration increased in all but the Davis summer foraging range, but was statistically significant at Mawson only (S2 File).

Associations between population growth and environmental change

Regional population growth was significantly correlated with three covariates with a five-year time lag: a negative correlation with increasing summer air temperature in the breeding areas (r = -0.638, p = 0.008, S3 File, Fig 6), and negative correlations with increasing sea-ice cover and decreasing air temperature in the winter foraging regions (r = -0.753, p = 0.008; r = -0.854, p = 0.004, respectively, S3 File, Fig 6). As air temperature and sea-ice cover in the winter foraging regions are themselves significantly correlated (S2 File), the latter two associations may reflect a response to the same environmental process or linkage. There were no significant correlations between regional population change and un-lagged covariate data (S3 File).

Discussion

Our synthesis of spatially and temporally extensive population data across EA has revealed a broad-scale and consistent increase in Adélie penguin populations over the last 50 years. This contrasts strongly with the WAP where, notwithstanding regional variation, overall Adélie populations have decreased over the last three decades [15,17]. These contrasting fortunes on opposite sides of the continent have occurred against a backdrop of strong change in the physical environmental in the WAP and apparent weaker change in EA. An intriguing outcome from these trans-Antarctic population studies is that despite the weaker change in the physical environment in EA, the magnitude of Adélie penguin population change in EA is similar to or greater than in the WAP (1.9±0.5% increase yr^{-1} in EA: this study; 1.1±0.8% decrease yr^{-1} in WAP: [15]).

Our challenge is to identify the conditions which either promoted or allowed the EA Adélie penguin population to increase over the last half century when data on many ecosystem components and processes are lacking. Such substantial population increases are possible through broad-scale movement of individuals to EA from elsewhere, or increases in key demographic parameters such as reproduction or survival in the EA populations. While genetic studies indicate that large-scale dispersal has occurred over geological time-frames in the past [37], Adélie penguins generally show strong natal site return over decadal time-frames. Environmental perturbations can induce movement between colonies, but movement within decadal time-frames has only been observed over relatively local spatial scales [38,39]. We therefore propose that

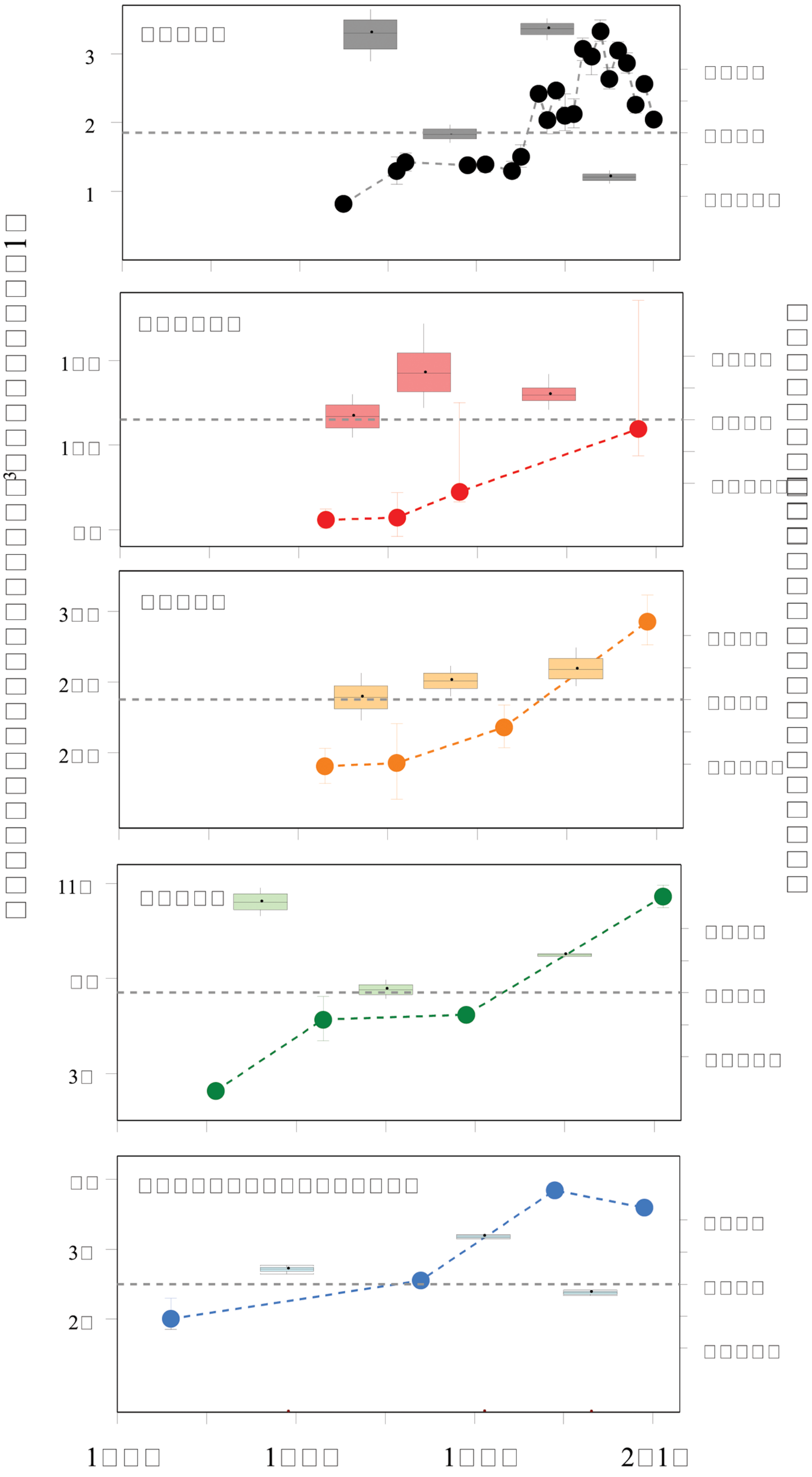

Fig 4. Change in Adélie penguin breeding population size at five regional populations over the last 35–54 years. Population changes are represented by time series of abundance estimates (solid circles with error bars) and boxplot summaries of population growth rates between estimates. Values are medians and 95% percentile limits. Grey dashed lines indicate zero growth rate.

doi:10.1371/journal.pone.0139877.g004

the most likely explanation for the increasing populations across EA over the last 30–50 years is related to changes in key demographic parameters such as survival and reproductive success in response to improved environmental or ecological conditions.

A key finding of our study is the stronger support for 5-year lagged associations between population growth and environmental change compared with immediate associations. This is consistent with results of local, detailed studies in EA, the WAP and the WRS which show or infer environmental conditions primarily influencing young penguins prior to recruiting into the breeding population when around 5 years old [22,29,40,41]. Our results identified a faster rate of population increase at the regional population scale five years after periods when winter sea-ice cover decreased, consistent with findings from local studies with more frequent observations [22,29]. Because direct observations of interactions between Adélie penguins and winter sea-ice are extremely difficult, explanations about the precise link between sea-ice cover and survival or population growth are necessarily speculative. Some proposed mechanisms include increased foraging south of the southern boundary of the Antarctic Circumpolar Current where prey is more available when sea-ice is less extensive or consolidated [22], more and larger leads in the pack-ice improving access between foraging and resting habitats, and decreased spatial overlap with leopard seals leading to lower predation rates [22,41].

Polynyas (predictable areas of open water within the sea-ice) are widely considered to be important for the presence and persistence of Adélie penguin breeding colonies [22,27] but their importance as drivers or constraints of population growth has received little attention. The finding of a positive relationship between regional Adélie penguin population size and

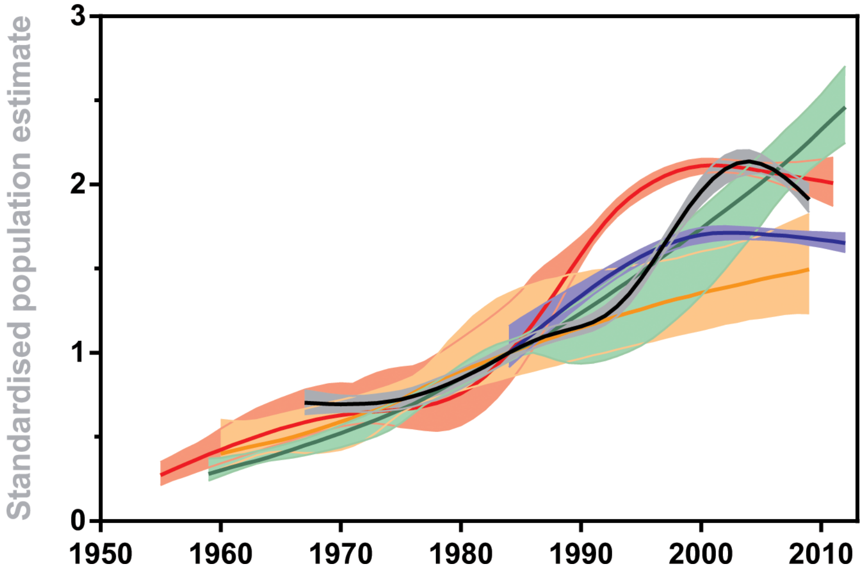

Fig 5. Change in local Adélie penguin populations over the last 50–60 years. The sites shown are those with the richest time series at each regional population (black: Rumpa Island, Syowa; red: Béchervaise Island, Mawson; orange: Gardner Island, Davis; green: Whitney Point, Casey; blue: Petrel Island, Dumont d'Urville). The time series were standardized for each site to a common population estimate for the 1984/85 breeding season (the earliest year with data across all sites) and fitted with generalized additive models

doi:10.1371/journal.pone.0139877.g005

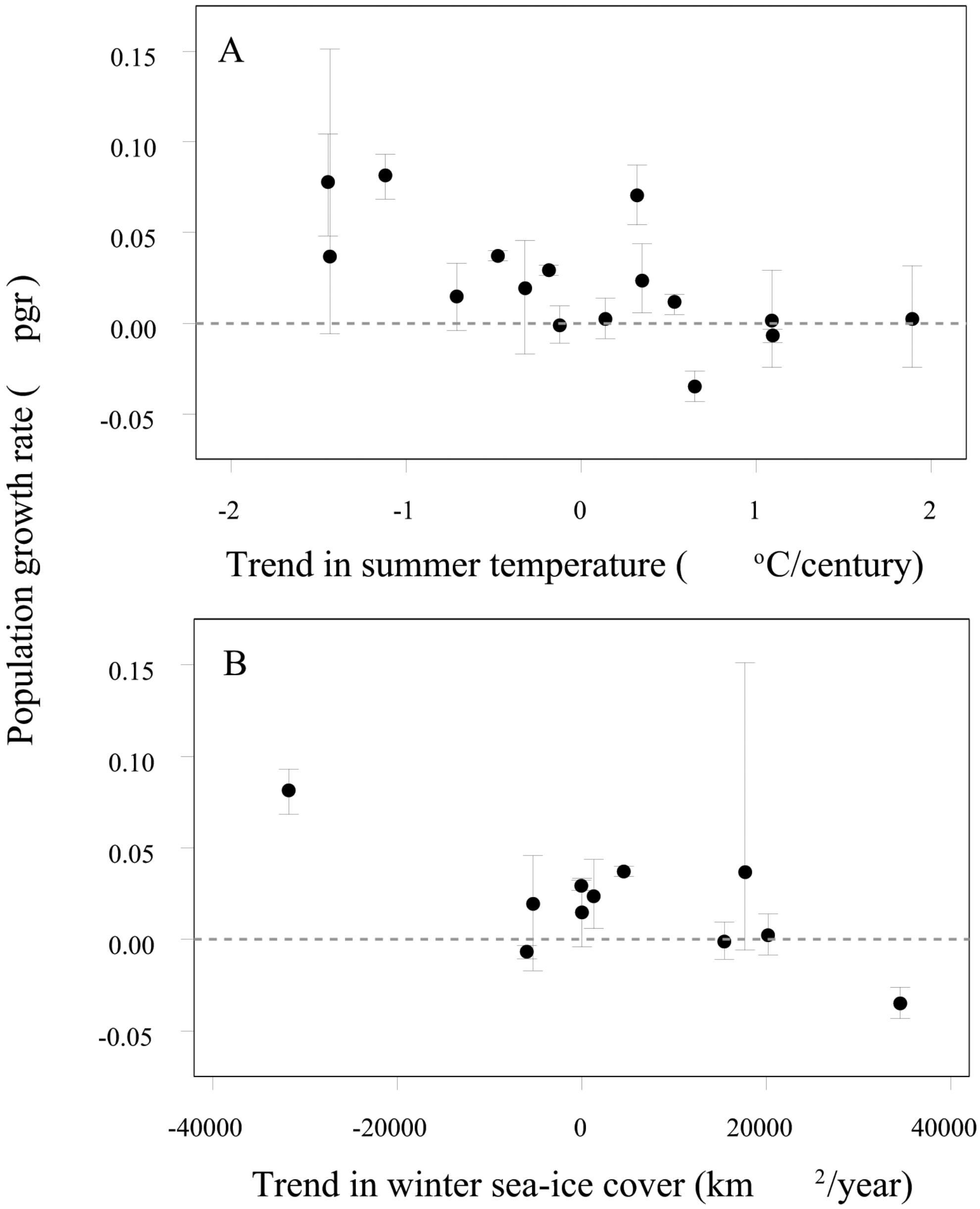

Fig 6. Associations between regional Adélie penguin population growth rate and change in environmental covariates. (A) Trend in summer air temperature at breeding regions lagged by five years; (B) Trend in winter sea-ice cover in winter foraging regions lagged by five years.

doi:10.1371/journal.pone.0139877.g006

primary productivity (mg C m^{-2}) of the nearest polynya by [42] suggests a causal linkage to unit area productivity, polynya size (since productivity and polynya size are correlated [42]), or total polynya productivity (= productivity x size). The locations of summer foraging regions for the five regional populations (Fig 2) correspond closely with polynyas identified by [42]. We found only limited changes in measured physical covariates in the summer foraging regions and no relationships between population changes and physical changes. We could not test for an association between population change and primary productivity change in the same manner as for other physical covariates because existing primary productivity time series are too short. However, in lieu of an analysis including long-term productivity data, we found

no association between our estimates of multi-decadal regional population change and 5-year (1997–2002) average values for primary productivity, polynya size and total productivity for the polynyas closest to each population. A recent more local study in the Ross Sea similarly found no relationship between polynya productivity and Adélie penguin reproductive parameters over a 13-year period, leading the authors to conclude that a link between primary productivity and Adélie penguin population dynamics may only be applicable at larger spatial and temporal scales [43]. Given our spatially and temporally extensive study also provides no support for this widely hypothesized phenomenon, we concur with the authors of [43] that any causal link between polynyas and penguin population dynamics should be considered with caution until more definitive evidence is available.

We found population growth was also associated with changes in physical conditions at the summer breeding areas. The negative association between lagged regional population growth and trend in air temperature in the summer breeding areas could be a manifestation of both direct and indirect processes affecting reproductive success. Potential effects of increasing air temperatures on reproductive success include reduced chick survival or condition through increased thermoregulatory stress [44], or increased nest or chick failure through increased snow melt and consequent flooding of nests in poorly drained areas [45]. The network of remotely operating cameras used to standardize population counts will provide the detailed data required to investigate the association between reproductive success and snow fall and melt across EA regional populations [36].

While variations in environmental conditions such as sea-ice and air temperature may have influenced or mediated decadal-scale variation in population growth across EA, explanations for the apparent widespread, long-term increase since the first population counts in the 1960s may be related to events that occurred before or at the time that population and environmental data started to become available. Two aspects of the EA marine environment have changed at a broad spatial scale and at a time that may provide a link to the long-term population increase. First, harvesting of baleen whales, krill and fish across EA waters through the 20th century [46,47] could have altered the food web dynamics and reduced inter-specific competition for food. Second, a proposed circumpolar contraction of sea-ice extent in the mid-20th century [48], including across EA waters [49], may have been beneficial to Adélie penguins. The proposed contraction of winter sea-ice area by 20–30% has been contentious because it relies on whaling ship locations as a proxy for the sea-ice edge [50,51]. However, it is supported by independent evidence from regional ice core studies in EA [52], the criticisms have been addressed in subsequent regional analyses of whaling data [49] that have not been re-challenged, and the proposed contractions in the Ross and Weddell Seas have been further supported by analysis of whaling [53] and ice core data [54], so it remains a plausible proposition in EA and elsewhere. A link between mid 20th century sea-ice contraction and positive population growth in EA remains speculative because it cannot be rigorously assessed due to the absence of historical population data from EA in first half of the 20th century. However, the time when sea-ice is estimated to have contracted to minimum levels (the early 1970s) [48] coincides with the timing of the early population time series data, and it would be consistent with the optimal sea-ice habitat model of [6] for populations subject to sea-ice above optimal levels. For Adélie penguins in EA where sea-ice is extensive, a causal link could be through increased access to prey as extensive sea-ice contracted [22]. Accessibility to prey is important to land-breeding predators such as Adélie penguins because this activity is a major component of their energy budget. On a circumpolar scale, the contrasting response of Adélie populations to environmental change in east and west Antarctica is consistent with the hypothesis of a non-linear response to decreasing sea-ice (for EA in the mid-20th century, for the WAP in the last 30 years).

Despite relatively consistent population increases across regional populations, our finding of variation within each regional population indicates that local processes affect population growth in addition to regional processes. The recent plateauing or decrease in some local Adélie populations may indicate density-dependent processes beginning to take effect after prolonged increases. Two possible density-dependent processes which are important for Adélie penguins include prey depletion in the summer foraging ranges and limits to breeding habitat. It is unclear whether predator-mediated prey depletion would impact all populations in a regional population simultaneously or not. This depends on whether there is geographic separation of foraging ranges for neighbouring local populations [55]. Density-dependent limits on breeding habitat could occur at different times at local sites within a regional population because breeding sites can differ substantially in their areas of suitable habitat, and this could result in increased spatial heterogeneity in population growth between local sites.

The Adélie penguin is not the only higher-order predator currently increasing in EA. Humpback whales (*Megaptera novaeangliae*) feeding off EA have been increasing by up to 13% yr^{-1} since 1978, and appear to be increasing at a faster rate than populations feeding off the WAP and Amundsen-Bellingshausen Seas [56]. In contrast, populations of winter breeding Emperor penguins (*Aptenodytes forsteri*) have declined at several sites across EA over the last 30–40 years [57,58]. Data from multiple species with different ecologies and life histories are necessary to provide robust insights into ecosystem processes, but finding parsimonious explanations for varying trends in these higher-order predators is difficult.

Current advances in remote, large-scale observation of Southern Ocean higher-order predators show promise for improved spatially extensive monitoring of land-breeding predators into the future [31,59–63]. Similar advances for assessing and monitoring the less visible components of marine ecosystems at lower trophic levels would help enormously in explaining population trends for multiple higher-order predators and for understanding the linkages from the physical environment up to top predators. It will be important that new technologies and methods are rigorously standardized with historical and current methods to ensure interpretation of ecological change is not confounded by changing methodology. For example, differing conclusions about Adélie population change in EA from our study compared with those from a recent global assessment of Adélie populations [31] may be a consequence of differing levels of standardization. The global study compared contemporary satellite-based EA population estimates, which were calibrated against direct counts at sites in the WAP and WRS [64], with published EA historical estimates. The study concluded from this comparison that populations had increased in the Dumont d'Urville region, had mixed increases and decreases in the Casey and Mawson regions, and decreased in the Syowa and Davis regions, whereas we found regionally consistent increases across EA. The different conclusions could result from two aspects of standardization. First, we re-constructed historical estimates using the same standardization data and adjustment process as for our contemporary estimates, while [31] used published historical estimates that have recently been shown to have unrecognized biases [32]. Second, our contemporary direct estimates are substantially higher than satellite estimates in [31] for the same locations and similar years, suggesting the satellite calibration using WAP and WRS data may not be representative of conditions in EA. The spatially extensive, direct, standardized data from this study will provide a valuable basis for calibrating satellite estimation of EA Adélie penguin populations in the future.

The future global status of Adélie penguins and other higher-order predators will depend on the complex interplay between the bottom-up effects of a changing physical environment and the top-down effects of human activities such as fishing and tourism. Qualitative models predict that most Adélie penguin populations across EA will decrease or could disappear in response to tropospheric temperatures rising 2°C above pre-industrial levels [27]. The sea-ice

optimum model proposed by [6] infers that while decreases in the frequency of heavy sea-ice years would result in differential growth rates for different populations depending on the sea-ice conditions of each population relative to the optimum, eventual declines are expected across all populations as sea-ice decreases further. Given the large spatial scales over which penguins interact with their environment, particularly during the winter months, we would expect all local populations within each EA regional population to be at a similar position along the frequency of heavy ice years axis of the sea-ice optimum curve. The fact that local populations within each regional population show varying trends indicates the importance of driving factors in addition to sea-ice being important for population growth. Consequently, studies quantifying penguin responses over large spatial and temporal scales and across wide environmental domains, such as the work presented here, are critical for understanding ecosystem change and improving predictions of future changes in Adélie penguin populations in a changing environment.

Supporting Information

S1 File. Additional details of population count data.
(DOC)

S2 File. Additional details of environmental covariates.
(DOC)

S3 File. Results of Pearson product-moment correlations between population growth and trends in environmental covariates.
(DOC)

Acknowledgments

This work was supported by the Australian Antarctic Program (recently under AAS projects 2722, 4087 and 4088), the Japanese Antarctic Research Expeditions (recently under programs AMB1 and AP12), and the Institut Paul Emile Victor (IPEV program 109), Zone Atelier Antarctique (CNRS-INEE), and Terres Australes et Antarctiques Françaises. Numerous expeditioners assisted with population counts and servicing or downloading cameras. Andrew Klekociuk provided ERA-Interim reanalysis data. David Smith and Matthew Oakes produced the figures. The comments of two reviewers improved the manuscript.

Author Contributions

Conceived and designed the experiments: CS LE AT AK CB KD HW. Performed the experiments: CS LE AT AK CB KD HW. Analyzed the data: CS LE. Contributed reagents/materials/analysis tools: JM KN. Wrote the paper: CS LE AT AK CB KD HW.

References

1. Stammerjohn SE, Martinson DG, Smith RC, Yuan X, Rind D (2008) Trends in Antarctic annual sea ice retreat and advance and their relation to El Nino-Southern Oscillation and Southern Annular Mode variability. J Geophys Res 113: C03S90.
2. Holland PR, Kwok R (2012) Wind-driven trends in Antarctic sea-ice drift. Nat Geosci 5: 872–875
3. Parkinson CL (2004) Southern Ocean sea ice and its wider linkages: insights revealed from models and observations. Antarct Sci 16: 387–400.
4. Massom RA, Stammerjohn SE (2010) Antarctic sea ice change and variability—physical and ecological implications. Polar Sci 4: 149–186.
5. Loeb V, Siegel V, Holm-Hansen O, Hewitt R, Fraser W, Trivelpiece W, et al. (1997) Effects of sea-ice extent and krill or salp dominance on the Antarctic food web. Nature 387: 897–900.

6. Smith RC, Ainley D, Baker K, Domack E, Emslie S, Fraser B, et al. (1999) Marine ecosystem sensitivity to climate change. Bioscience 49: 393–404.
7. Fraser WR, Hofmann EE (2003). A predator's perspective on causal links between climate change, physical forcing and ecosystem response. Mar Ecol Prog Ser 265: 1–15.
8. Moline MA, Claustre H, Frazer TK, Schofield O, Vernet M (2004) Alteration of the food web along the Antarctic Peninsula in response to a regional warming trend. Glob Change Biol 10: 1973–1980.
9. Murphy EJ, Trathan PN, Watkins JL, Reid K, Meredith MP, Forcada J, et al. (2007) Climatically driven fluctuations in Southern Ocean ecosystems. P R Soc B 274: 3057–3067.
10. Ducklow HW, Baker K, Martinson DG, Quetin LB, Ross RM, Smith RC, et al. (2007) Marine pelagic ecosystems: the West Antarctic Peninsula. Philos T Roy Soc B 362: 67–94.
11. Emmerson L, Southwell C (2008) Sea ice cover and its influence on Adélie penguin reproductive performance. Ecology 89: 2096–2102. PMID: 18724720
12. Kato A, Yoshioka A, Sato K (2009) Foraging behaviour of Adélie penguins during incubation period in Lützow-Holm Bay. Polar Biol 32: 181–186.
13. Reid K, Croxall JP (2001) Environmental response of upper-level predators reveals a system change in an Antarctic marine ecosystem. Proc R Soc London, Ser B 268: 377–384.
14. Forcada J, Trathan PN, Reid K, Murphy EJ (2005) The effects of global climate variability in pup production of Antarctic fur seals. Ecology 86: 2408–2417.
15. Lynch HJ, Naveen R, Trathan PN, Fagan WF (2012) Spatially integrated assessment reveals widespread changes in penguin populations on the Antarctic Peninsula. Ecology 93: 1367–1377. PMID: 22834377
16. Lyver POB, Barron M, Barton KJ, Ainley DG, Pollard A, Gordon S, et al. (2014) Trends in the breeding population of Adélie penguins in the Ross Sea, 1981–2012: a coincidence of climate and resource extraction effects. PLoS ONE 9: e91188. doi: 10.1371/journal.pone.0091188 PMID: 24621601
17. Trivelpiece WZ, Hinke JT, Miller AK, Reiss CS, Trivelpiece SG, Watters GM (2011) Variability in krill biomass links harvesting and climate warming to penguin population changes in Antarctica. P Natl Acad Sci USA 108: 7625–7628.
18. Sailley SF, Ducklow HW, Moeller HV, Fraser WR, Schofield OM, Steinberg DK, et al. (2013) Carbon fluxes and pelagic ecosystem dynamics near two western Antarctic Peninsula Adélie penguin colonies: an inverse model approach. Mar Ecol Prog Ser 492: 253–272.
19. Massom R, Reid P, Stammerjohn S, Raymond B, Fraser A, Ushio S (2013) Change and variability in east Antarctic sea ice seasonality, 1979/80-2009/10. PLoS ONE 8: e64756. doi: 10.1371/journal.pone.0064756 PMID: 23705008
20. Atkinson A, Siegel V, Pakhomov E, Rothery P (2004) Long-term decline in krill stock and increase in salps within the Southern Ocean. Nature 432: 100–103. PMID: 15525989
21. Stonehouse B (1965) Counting Antarctic animals. New Sci 29: 273–276.
22. Wilson PR, Ainley DG, Nur N, Jacobs SS, Barton KJ, Ballard G, et al. (2001) Adélie penguin population change in the pacific sector of Antarctica: relation to sea-ice extent and the Antarctic Circumpolar Current. Mar Ecol Prog Ser 213: 301–309.
23. Lynch H J, Fagan WF, Naveen R, Trivelpiece SG, Trivelpiece WZ (2009) Timing of clutch initiation in *Pygoscelis* penguins on the Antarctic Peninsula: towards an improved understanding of off-peak census correction factors. CCAMLR Sci 16: 149–165.
24. Kato A, Ropert-Coudert Y, Naito Y (2002) Changes in Adélie penguin breeding populations in Lützow-Holm Bay, in relation to sea-ice conditions. Polar Biol 25: 934–938.
25. Weimerskirch H, Inchausti P, Guinet C, Barbraud C (2003) Trends in bird and seal populations as indicators of a system shift in the Southern Ocean. Antarct Sci 15: 249–256.
26. Ainley DG, Clarke ED, Arrigo K, Fraser WR, Kato A, Barton KJ et al. (2005) Decadal-scale changes in the climate and biota of the Pacific sector of the Southern Ocean, 1950s to the 1990s. Antarct Sci 17: 171–182.
27. Ainley D, Russell J, Jenouvrier S, Woehler E, Lyver POB, Fraser WR, et al. (2010) Antarctic penguin response to habitat change as Earth's troposphere reaches 2°C above preindustrial levels. Ecol Monogr 80: 49–66.
28. Kato A, Ropert-Coudert Y (2006) Rapid increase in Adélie penguin populations in the Lützow-Holm Bay area since the mid 1990s. Polar Biosci 20: 55–62.
29. Jenouvrier S, Barbraud C, Weimerskirch H (2006) Sea ice affects the population dynamics of Adélie penguins in Terre Adélie. Polar Biol 29: 413–423.

30. Woehler EJ, Cooper J, Croxall JP, Fraser WR, Kooyman GL, Miller GD, et al. (2001) A statistical assessment of the status and trends of Antarctic and Subantarctic seabirds. Cambridge: SCAR, Scott Polar Research Institute. 43 p.
31. Lynch HJ, LaRue MA (2014) First global census of the Adélie penguin. The Auk 131: 457–466.
32. Southwell C, Emmerson L, Newbery K, McKinlay J, Kerry K, Woehler E, et al. (2015) Re-constructing historical Adélie penguin abundance estimates by retrospectively accounting for detection bias. PLoS ONE 10: e0123540. doi: 10.1371/journal.pone.0123540 PMID: 25909636
33. McKinlay J, Southwell C, Trebilco R (2010) Integrating Count Effort by Seasonally Correcting Animal Population Estimates (ICESCAPE): A method for estimating abundance and its uncertainty from count data using Adélie penguins as a case study. CCAMLR Sci 17: 213–227.
34. Southwell C, McKinlay J, Emmerson L, Trebilco R, Newbery K (2010) Improving estimates of Adélie penguin breeding population size: developing factors to adjust one-off population counts for availability bias. CCAMLR Sci 17: 229–241.
35. Southwell C, McKinlay J, Low M, Wilson D, Newbery K, Lieser J, et al. (2013) New methods and technologies for regional-scale abundance estimation of land-breeding marine animals: application to Adélie penguin populations in East Antarctica. Polar Biol 36: 843–856.
36. Southwell C, Emmerson L (2015) Remotely-operating camera network expands Antarctic seabird observations of key breeding parameters for ecosystem monitoring and management. J Nat Conserv 23: 1–8.
37. Millar CD, Subramanian S, Heupink TH, Swaminathan S, Baroni C, Lambert DM (2012) Adélie penguins and temperature changes in Antarctica: a long-term view. Integr Zool 7: 113–120. doi: 10.1111/j.1749-4877.2012.00288.x PMID: 22691195
38. Dugger KM, Ainley DG, Lyver POB, Barton KJ, Ballard G (2010) Survival differences and the effect of environmental instability on breeding dispersal in an Adélie penguin meta-population. P Natl Acad Sci USA 107: 12375–12380.
39. LaRue MA, Ainley DG, Swanson M, Dugger KM, Lyver PO'B, Barton K, et al. (2013) Climate change winners: receding ice fields facilitate colony expansion and altered dynamics in an Adélie penguin metapopulation. PLoS ONE 8: e60568. doi: 10.1371/journal.pone.0060568 PMID: 23573267
40. Hinke JT, Salwicka K, Trivelpiece SG, Watters GM, Trivelpiece WZ (2007) Divergent responses of *Pygoscelis* penguins reveal a common environmental driver. Oecologia 153: 845–855. PMID: 17566778
41. Emmerson L, Southwell C (2011) Adélie penguin survival: age structure, temporal variability and environmental influences. Oecologia 167: 951–965. doi: 10.1007/s00442-011-2044-7 PMID: 21691857
42. Arrigo KR, van Dijken GL (2003) Phytoplankton dynamics within 37 Antarctic coastal polynya systems. J Geophys Res 108: 3271.
43. Dugger KM, Ballard G, Ainley DG, Lyver PO'B, Schine C (2014) Adélie penguins coping with environmental change: results from a natural experiment at the edge of their breeding range. Front Ecol Evol 2: 68.
44. Chappell MA, Morgan KR, Bucher TL (1990) Weather, microclimate, and energy costs of thermoregulation for breeding Adélie penguins. Oecologia 83: 420–426.
45. Bricher PK, Lucieer A, Woehler EJ (2008) Population trends of Adélie penguin (*Pygoscelis adeliae*) breeding colonies: a spatial analysis of the effects of snow accumulation and human activities. Polar Biol 31: 1397–1407.
46. Kock K-H (2007) Antarctic marine living resources—exploitation and its management in the Southern Ocean. Antarct Sci 19: 231–238.
47. Ainley DG, Blight LK (2009) Ecological repercussions of historical fish extraction from the Southern Ocean. Fish Fish 10: 13–38.
48. de la Mare WK (1997) Abrupt mid-twentieth-century decline in Antarctic sea-ice extent from whaling records. Nature 389: 57–60.
49. de la Mare WK (2009) Changes in Antarctic sea-ice extent from direct historical observations and whaling records. Climate Change 92: 461–493.
50. Vaughan S (2000) Can Antarctic sea ice extent be determined from whaling records? Polar Rec 36: 345–347.
51. Ackley S, Wadhams P, Comiso JC, Worby A (2003) Decadal decrease of Antarctic sea ice extent inferred from whaling records revisited on the basis of historical and modern sea ice records. Polar Res 22: 19–25.
52. Curran MAJ, van Ommen TD, Morgan VI, Phillips KL, Palmer AS (2003) Ice core evidence for Antarctic sea ice decline since the 1950s. Science 202: 1203–1206.

53. Cotte C, Guinet C (2007) Historical whaling records reveal major regional retreat of Antarctic sea ice. Deep-Sea Res Pt I 54: 243–252.

54. Sinclair KE, Bertler NAN, Bowen MM, Arrigo KR (2014) Twentieth century sea-ice trends in the Ross Sea from a high-resolution, coastal ice-core record. Geophys Res Lett 41: 3510–3516.

55. Ainley DG, Ribic CA., Ballard G, Heath S, Gaffney B, Karl J, et al. (2004) Geographic structure of Adélie penguin populations: size, overlap and use of adjacent colony-specific foraging areas. Ecol Monogr 74: 159–178.

56. Branch TA (2011) Humpback whale abundance south of 60°S from three complete circumpolar sets of surveys. J Cet Res Manage 3: 53–69.

57. Barbraud C, Gavrilo M, Mizin Y, Weimerskirch H (2012) Comparison of emperor penguin declines between Pointe Géologie and Haswell Island over the past 50 years. Antarct Sci 23: 461–468.

58. Robertson G, Wienecke B, Emmerson L, Fraser AD (2014) Long-term trends in the population size and breeding success of emperor penguins at the Taylor Glacier colony, Antarctica. Polar Biol 37: 251–259.

59. Fretwell PT, Trathan PN (2009) Penguins from space: faecal stains reveal the location of emperor penguin colonies. Global Ecol Biogeogr 18: 543–552.

60. Fretwell PT, LaRue MA, Morin P, Kooyman GL, Wienecke B, Ratcliffe N, et al. (2012) An emperor penguin population estimate: the first global, synoptic survey of a species from space. PLoS ONE 7: 1–11.

61. Schwaller M, Southwell C, Emmerson L (2013) Continental scale mapping of Adélie penguin colonies from Landsat imagery. Remote Sens Environ 139: 353–364.

62. Lynch HJ, Schwaller MR (2014) Mapping the abundance and distribution of Adélie penguins using Landsat–7: first steps towards an integrated multi-sensor pipeline for tracking populations at the continental scale. PLoS ONE 9:e113301. doi: 10.1371/journal.pone.0113301 PMID: 25412466

63. LaRue MA, Rotella JJ, Garrott RA, Siniff DB, Ainley DG, Stauffer GE, et al. (2011) Satellite imagery can be used to detect variation in abundance of Weddell seals (*Leptonychotes weddellii*) in Erebus Bay, Antarctica. Polar Biol 34: 1727–1737.

64. LaRue MA, Lynch HJ, Lyver PO'B, Barton K, Ainley DG, Pollard A, et al. (2014) A method for estimating colony sizes of Adélie penguins using remote sensing imagery. Polar Biol 37: 507–517.
